# Genome-wide characterization of the wall-associated kinase-like (WAKL) family in sesame (*Sesamum indicum*) identifies a *SiWAKL6* gene involved in resistance to *Macrophomina Phaseolina*

**DOI:** 10.1186/s12870-023-04658-1

**Published:** 2023-12-07

**Authors:** Wenqing Yan, Peilin Hu, Yunxia Ni, Hui Zhao, Xintao Liu, Hengchun Cao, Min Jia, Baoming Tian, Hongmei Miao, Hongyan Liu

**Affiliations:** 1 Institute of Plant Protection, Henan Academy of Agricultural Sciences, Postgraduate T&R Base of Zhengzhou University, Zhengzhou, Henan 450002 China; 2 Key Laboratory of Specific Oilseed Crops Genomics of Henan Province, Zhengzhou, Henan 450002 China; 3 School of Agricultural Sciences, Zhengzhou University, Zhengzhou, Henan 450002 China; 4 The Shennong Laboratory, Zhengzhou, Henan 450002 China

**Keywords:** Sesame, WAKL, Gene family, *Macrophomina Phaseolina*, Resistance

## Abstract

**Background:**

Sesame charcoal rot caused by *Macrophomina phaseolina* is one of the most serious fungal diseases in sesame production, and threatens the yield and quality of sesame. *WAKL* genes are important in the plant response to biotic stresses by sensing and transmitting external signals to the intracellular receptor. However, there is still a lack about the *WAKL* gene family and its function in sesame resistance to *M. phaseolina*. The aim of this study was to interpret the roles of *WAKL* genes in sesame resistance to *M. phaseolina*.

**Results:**

In this study, a comprehensive study of the *WAKL* gene family was conducted and 31 *WAKL* genes were identified in the sesame genome. Tandem duplication events were the main factor in expansion of the *SiWAKL* gene family. Phylogenetic analysis showed that the sesame *SiWAKL* gene family was divided into 4 groups. *SiWAKL* genes exhibited different expression patterns in diverse tissues. Under *M. phaseolina* stress, most *SiWAKL* genes were significantly induced. Notably, *SiWAKL6* was strongly induced in the resistant variety “Zhengzhi 13”. Functional analysis showed that *SiWAKL6* was induced by salicylic acid but not methyl jasmonate in sesame. Overexpression of *SiWAKL6* in transgenic *Arabidopsis thaliana* plants enhanced their resistance to *M. phaseolina* by inducing the expression of genes involved in the salicylic acid signaling pathway and reconstructing reactive oxygen species homeostasis.

**Conclusions:**

Taken together, the results provide a better understanding of functions about *SiWAKL* gene family and suggest that manipulation of these *SiWAKL* genes can improve plant resistance to *M. phaseolina*. The findings contributed to further understanding of functions of *SiWAKL* genes in plant immunity.

**Supplementary Information:**

The online version contains supplementary material available at 10.1186/s12870-023-04658-1.

## Introduction

The plant cell wall, composed of cellulose, hemicellulose, pectin and a few structural proteins, is not only crucial in cell morphology but is also the first line of defense against pathogens. Receptor-like kinases (RLKs) are an important kinase family in the cell membrane, which contains various extracellular domains that are suitable for recognizing external signals, such as leucine-rich repeats, lectins, lysine motifs, and epidermal growth factor extracellular domains [1, 2]. Under multifarious stresses, RLKs can recognize extracellular signals and transmit them into cells, subsequently activating the expression of downstream transcription factors (TF), pathogenesis-related (PR) genes and plant hormone-related genes [3, 4]. In vascular plants, cell wall-associated receptor kinases (WAKs) containing the wall-associated receptor kinase galacturonan-binding (GUB-WAK-bind) domain and protein kinase domain (PKinase) are a subfamily of the RLK family [5]. WAKL is the only protein known to act as a direct link between the cell wall and the plasma membrane, which enables plants to sense changes in cell wall structure and rapidly initiate intracellular signal transduction processes and defense responses [6, 7].

Pro25, the first member of the WAKLs, was found to be closely related to the cell wall in *Arabidopsis thaliana*. Therefore, it was renamed cell wall-associated kinase 1 (WAK1), and the concept of a cell wall-associated receptor kinase was proposed [8]. Subsequently, a highly conserved family containing *WAK*1-5 and 22 *WAK-like* genes (*WAKLs*) in *A. thaliana* was discovered [9].

WAKLs are important in plant responses to environmental stress. *AtWAKL10* can be induced by nitric oxide in *A. thaliana*. *AtWAKL10* plays a positive role in basic defense, effect-triggering immunity and salt stress but is negative in response to drought [10]. The cell wall pectin of *Craterostigma plantagineum* could bind to CpWAK1 and regulate its growth under non-pressure conditions. However, during dehydration, *C. plantagineum* glycine-rich protein 1 interacts with Ca^2+^ and pectin to form a complex that can bind to CpWAK1 with high affinity. Finally, pectin perturbations in the cell wall during dehydration were sensed by CpWAK1, leading to activation of downstream dehydration signaling pathways [11]. WAKLs could mediate defense responses induced by chitin. For instance, the wheat *TaWAK7D* gene can be induced by chitin stimulation, which plays a positive role in plant defense against *Rhizoctonia cerealis* [12]. The *Rlm9* gene in *Brassica napus* supports its resistance to blackleg, in which the GUB-WAK-bind domain of Rlm9 is involved in the recognition of pathogens [13]. Hurni et al. showed that *Htn1*, a *WAK* gene in maize, was closely associated with resistance to maize leaf blight. The pathogen penetrates the cell wall, and produces cell wall fragments that can be recognized and sensed by Htn1, which in turn transmits the signal downstream and activates the expression of *PR* genes to enhance maize resistance to leaf blight [14]. Similarly, *TaWAK6* is involved in wheat resistance to leaf rust [15], and *TaWAK2* enhances wheat resistance to *Fusarium graminearum* by binding to pectin [16], which further highlights the importance of *WAKL* gene family in plant disease resistance. Nevertheless, some *WAKLs* negatively regulate plant disease resistance. For example, *ScWAK1* can induced hypersensitive response and the expression of ethylene-related genes, negatively regulating the defense against *Sporisorium scitamineum* in sugarcane [17]. The *Snn1* gene encoding *WAK* in wheat conferred plant susceptibility by responding to the toxin produced by *Stagonospora nodorum* and triggering cell death, thereby promoting the proliferation of fungi in wheat [18].

Importantly, an increasing number of studies have indicated that WAKLs are the junctions that regulate plant stress signaling and development in plants [19]. *Xa4*, encoding a *WAK* gene, can enhance the thickness of the cell wall and thus confer rice durable resistance to *Xanthomonas oryzae* without affecting rice yield, which is significant in rice breeding [20]. Similarly, it is proposed that *ZmWAK* is the hub of fine-tuning between maize growth and defense. *ZmWAK* promotes cell growth under normal conditions but protects plants when maize plants attacked by pathogens [21], suggesting that studying WAKL-mediated mechanisms to balance plant immunity and yield contributes to crop breeding.

Sesame (*Sesamum indicum* L.), widely cultivated in tropical and subtropical regions, is one of the most nutritious oil crops. Sesame seeds contain unsaturated fatty acids and various natural antioxidants, which are healthy to humans [22, 23]. Sesame charcoal rot caused by *Macrophomina phaseolina* is one of the most serious fungal diseases in sesame production, and threatens the yield and quality of sesame, which diminishes sesame production of 10–15% or even over 80% in serious cases. Furthermore, Although the *WAKL* gene family is important in plant biotic stresses, there is still a lack of systematic studies on the roles of *WAKL* gene family in the interaction between sesame and *M. phaseolina*. In this study, the *WAKL* family in sesame was identified and comprehensively analyzed on a genome-wide scale. In addition, this study reported that *SiWAKL6* was crucial in plant immunity, acting in the SA signaling pathway and ROS homeostasis. The results not only provided more insights into the classification and biological function of SiWAKLs, but also promoted the application of the *SiWAKL* genes in the molecular breeding of sesame resistance to *M. phaseolina*.

## Results

### Identification and phylogenetic analysis of the *SiWAKL* gene family

A total of 31 *WAKL* genes were exhaustively identified based on the *Sesamum indicum* genome and were named *SiWAKL1*-*SiWAKL31* according to their position on the chromosome (Additional file: Table S1). The prefix “Si” represents the species “*S. indicum*”. Bioinformatic analysis showed that the SiWAKL proteins in sesame contained 504 to 787 amino acids. Their molecular weights ranged from 57.3 to 86.62 kDa and their isoelectric points ranged from 5.37 to 8.83. Most SiWAKL proteins are stable, and the high aliphatic index implied that they localized to the cell membrane.

**Fig. 1:**
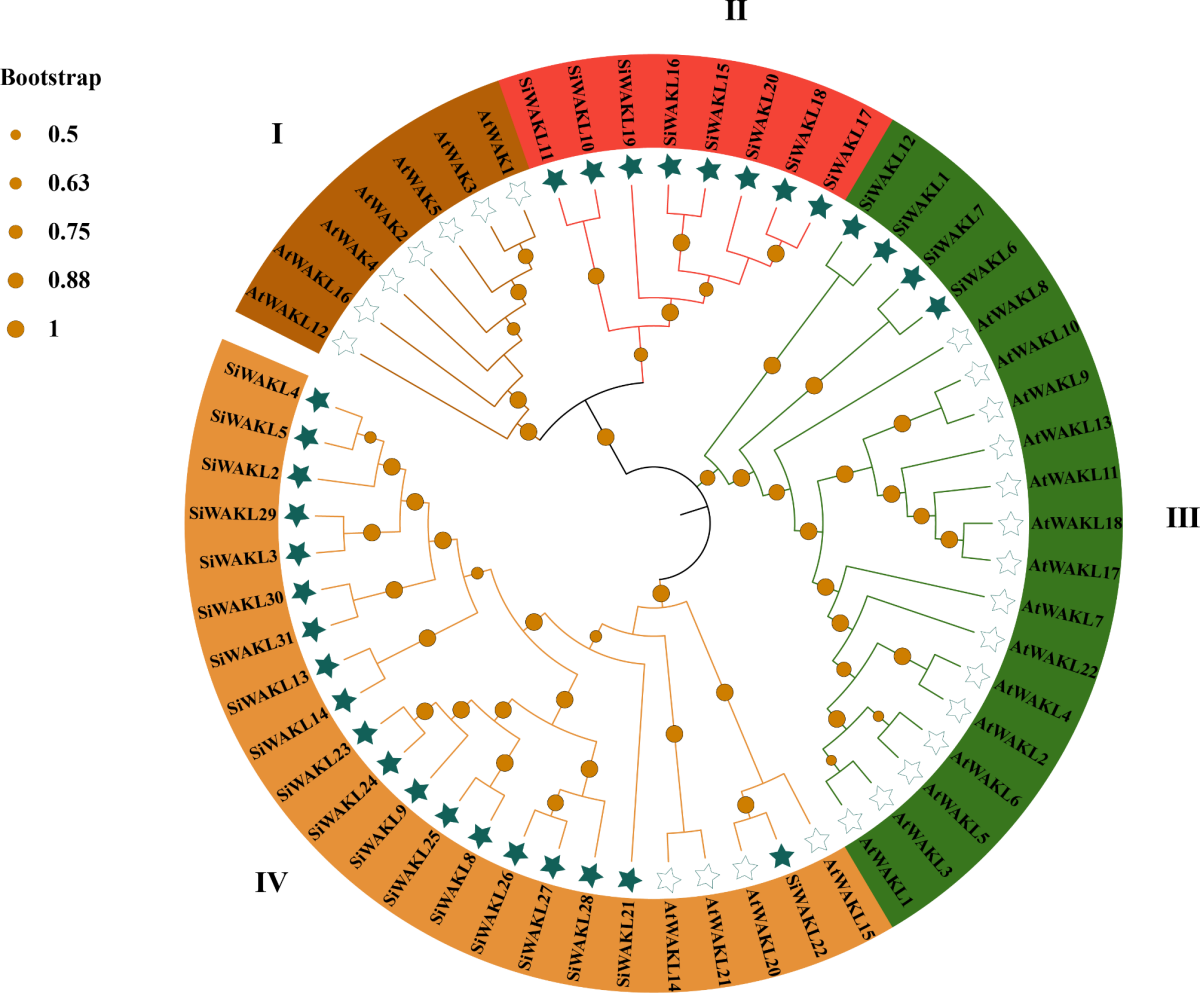
Phylogenetic analysis of the WAKL proteins in *S. indicum* and *A. thaliana.* Solid pentagrams represent SiWAKL proteins, while hollow pentagrams represent AtWAKL proteins

To investigate the taxonomic and evolutionary relationships of WAKL proteins in *S. indicum* and *A. thaliana*, a phylogenetic tree was constructed based on the aligned sequences of WAKL proteins. The WAKL proteins in *S. indicum* and *A. thaliana* were divided into 4 subfamilies, subfamilies I, II, III and IV (Fig. 1). Among them, subfamily IV is the largest group, containing 19 SiWAKL proteins and 4 AtWAKL proteins, followed by subfamily III, which contains 15 AtWAKL proteins and 4 SiWAKL proteins. Subfamily I contained only 7 AtWAKL proteins. Subfamily II contained no AtWAKL proteins, suggesting that WAKL proteins in subfamily II are conserved and unique WAKLs that have developed in *S. indicum* during evolution.

### Sequence analysis and functional prediction of SiWAKL proteins

**Fig. 2:**
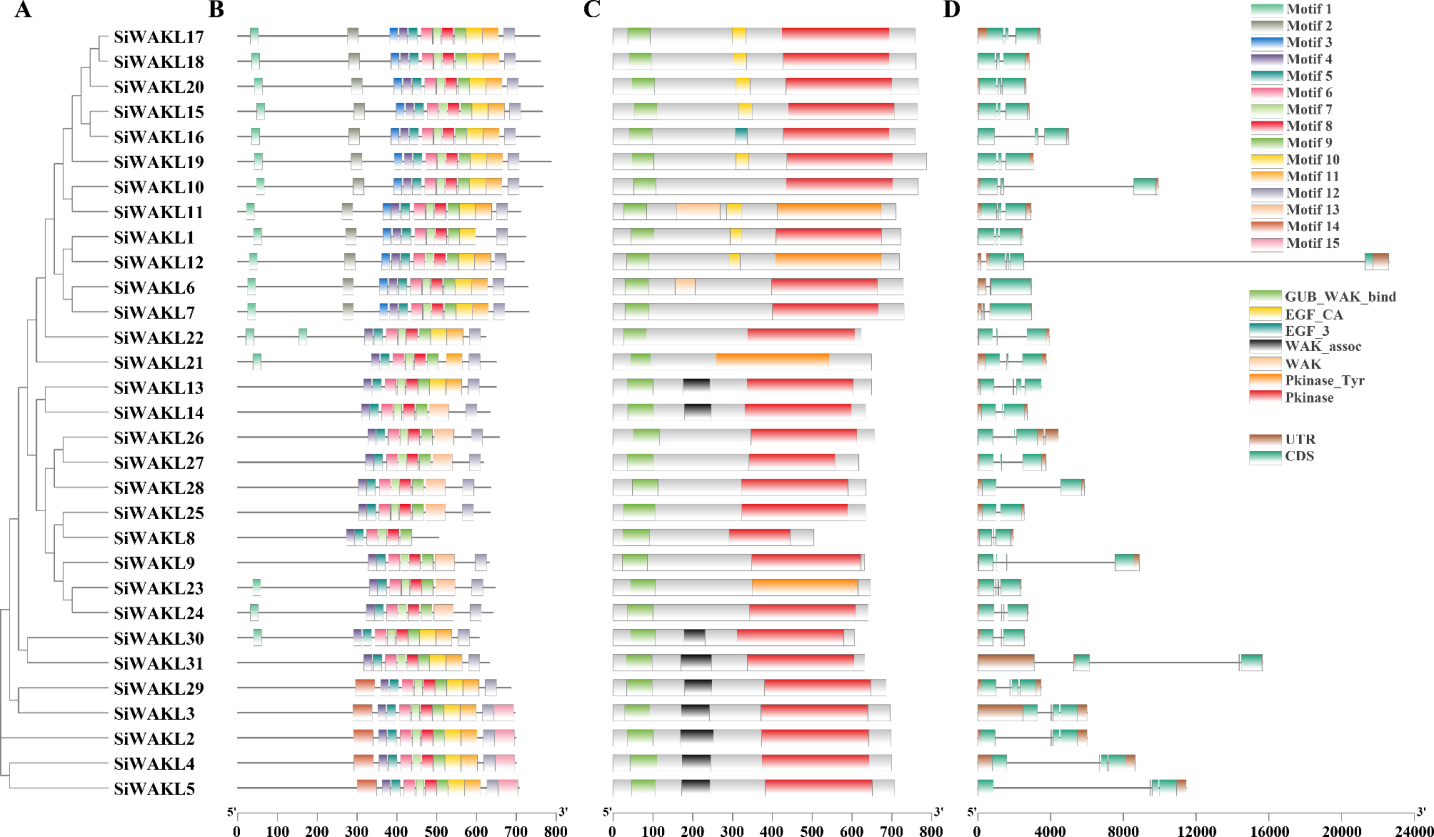
Sequence analysis of the *SiWAKL* gene family. Phylogenetic analysis (**A**), conserved motif (**B**), conserved domains (**C**) and gene structures (**D**) of the *SiWAKL* gene family

The evolutionary tree of the SiWAKLs proteins was constructed using MEGA7 software (Fig. 2A), which was similar to the results of the phylogenetic tree in Fig. 1, indicating the reliability of the results. Conserved motif analysis of SiWAKL proteins showed that all the SiWAKLs contain Motif 4, Motif 5, Motif 6, Motif 7, Motif 8 and Motif 9 in their C-terminal, indicating that the protein kinase domain was more conserved and important in the SiWAKLs (Fig. 2B). All SiWAKLs have GUB-WAK-bind domain in the N-terminal and protein kinase domain in C-terminal, which further demonstrates the reliability of the SiWAKL members in sesame (Fig. 2C). In addition, 11 members of SiWAKLs, including SiWAKL2, SiWAKL 3, SiWAKL 4, SiWAKL 5, SiWAKL 6, SiWAKL 11, SiWAKL 13, SiWAKL 14, SiWAKL 29, SiWAKL 30, and SiWAKL 31, had WAK or WAK-associated domains. A total of 8 SiWAKLs contained EGF-CA or EGF-3 domains (Fig. 2C). The exon‒intron structure showed that the exons of the *SiWAKL* genes ranged from 2 to 5, and most *SiWAKL* genes had 3 exons (Fig. 2D).

**Fig. 3:**
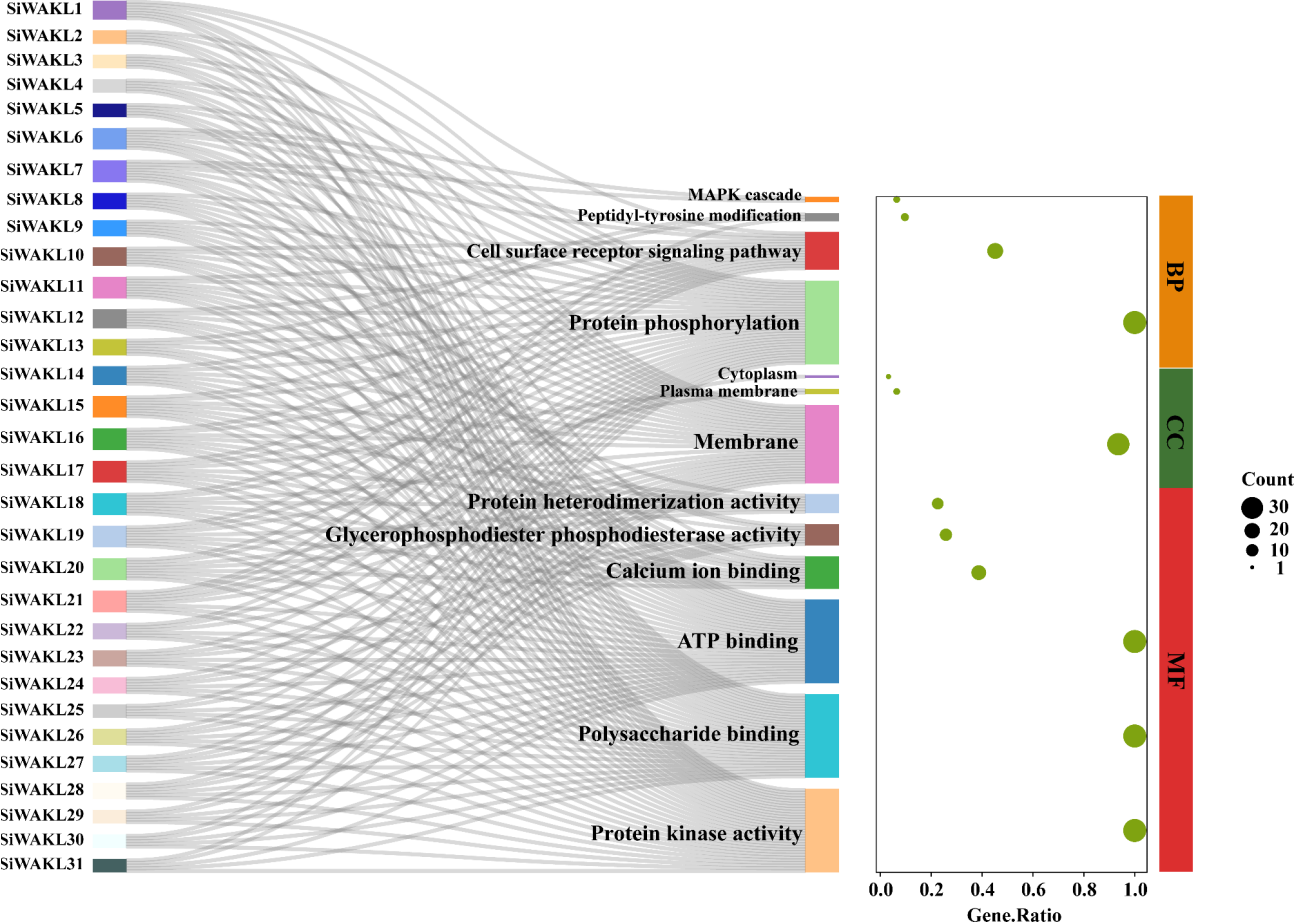
GO annotation of 31 SiWAKL proteins

To understand the biological roles of SiWAKLs initially, a functional prediction analysis was performed. Gene Ontology (GO) annotation was performed for 31 SiWAKLs based on biological process (BP), cellular component (CC) and molecular function (MF) terms (Fig. 3). In terms of BP, SiWAKL proteins mainly function in protein phosphorylation and the cell surface receptor signaling pathway, which are closely related to cellular perception and transduction of external signals. In terms of MF, it was demonstrated that SiWAKLs could bind polysaccharides, ATP and Ca^2+^, all of which have been shown to be associated with biotic and abiotic stresses in plants. Based on the GO annotation of SiWAKLs, it was inferred that SiWAKLs exerted protein phosphorylation and cell surface signal transduction by binding polysaccharides, ATP and Ca^2+^, which might be important for plant resistance to pathogens (Fig. 3).

### Chromosome localization and duplication events of *SiWAKL* genes

**Fig. 4:**
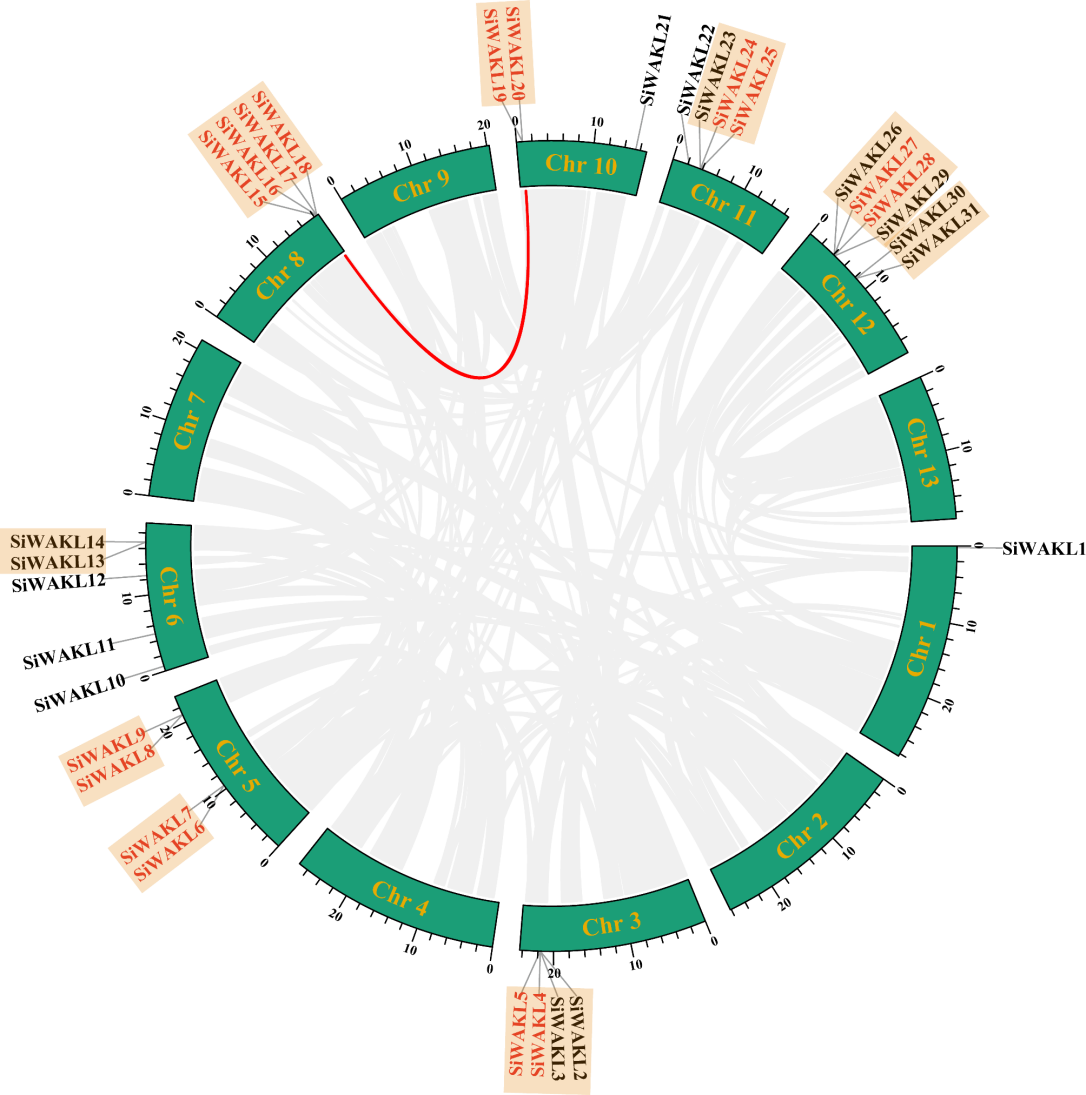
Chromosome localization and duplication events of *SiWAKL* genes. The green boxes represent 13 chromosomes of sesame. The gray lines indicate all the segmentally duplicated gene pairs within sesame genome, while red line highlight segmentally duplicated *SiWAKL* gene pairs *SiWAKL15* and *SiWAKL19*. *SiWAKL* genes under orange background represent gene clusters. *SiWAKL* genes in red font indicate tandemly duplicated *SiWAKL* genes

Based on the *S. indicum* genome, 31 *SiWAKL* genes were mapped unevenly to 8 chromosomes (Chr). *SiWAKL* genes were distributed on Chr 1, Chr 3, Chr 5, Chr 6, Chr 8, Chr 10, Chr 11 and Chr 12. Chr 12 contained the most *SiWAKL* genes (6 *SiWAKLs*, 19.4%), followed by Chr 6 (5 *SiWAKLs*, 16.1%). Chr 3, Chr 5, Chr 8 and Chr 11 all comprised 4 *SiWAKL* genes. In contrast, Chr 1 contained only one *SiWAKL* gene (Fig. 4). Additionally, 9 gene clusters were formed by 25 genes on seven chromosomes (Fig. 4).

Gene duplication events are important in the formation of new genes and plant adaptation. Segmental and tandem duplication are vital drivers in the expansion of gene families, especially plant RLK gene family [24]. To elucidate the mechanism of expansion of the *SiWAKL* gene family, synteny analysis of *SiWAKL* genes within the *S. indicum* genome was performed by MCScanX (Fig. 4). A total of 18 genes in sesame underwent duplication events, including 10 tandem duplication events formed by 18 *SiWAKL* genes and 1 segmental duplication event formed by 2 *SiWAKL* genes *SiWAKL15 and SiWAKL19* (Fig. 4), indicating that tandem duplication events are the prime driver of *SiWAKL* gene family expansion.

### Evolution analysis of *SiWAKL* genes in several plants

**Fig. 5:**
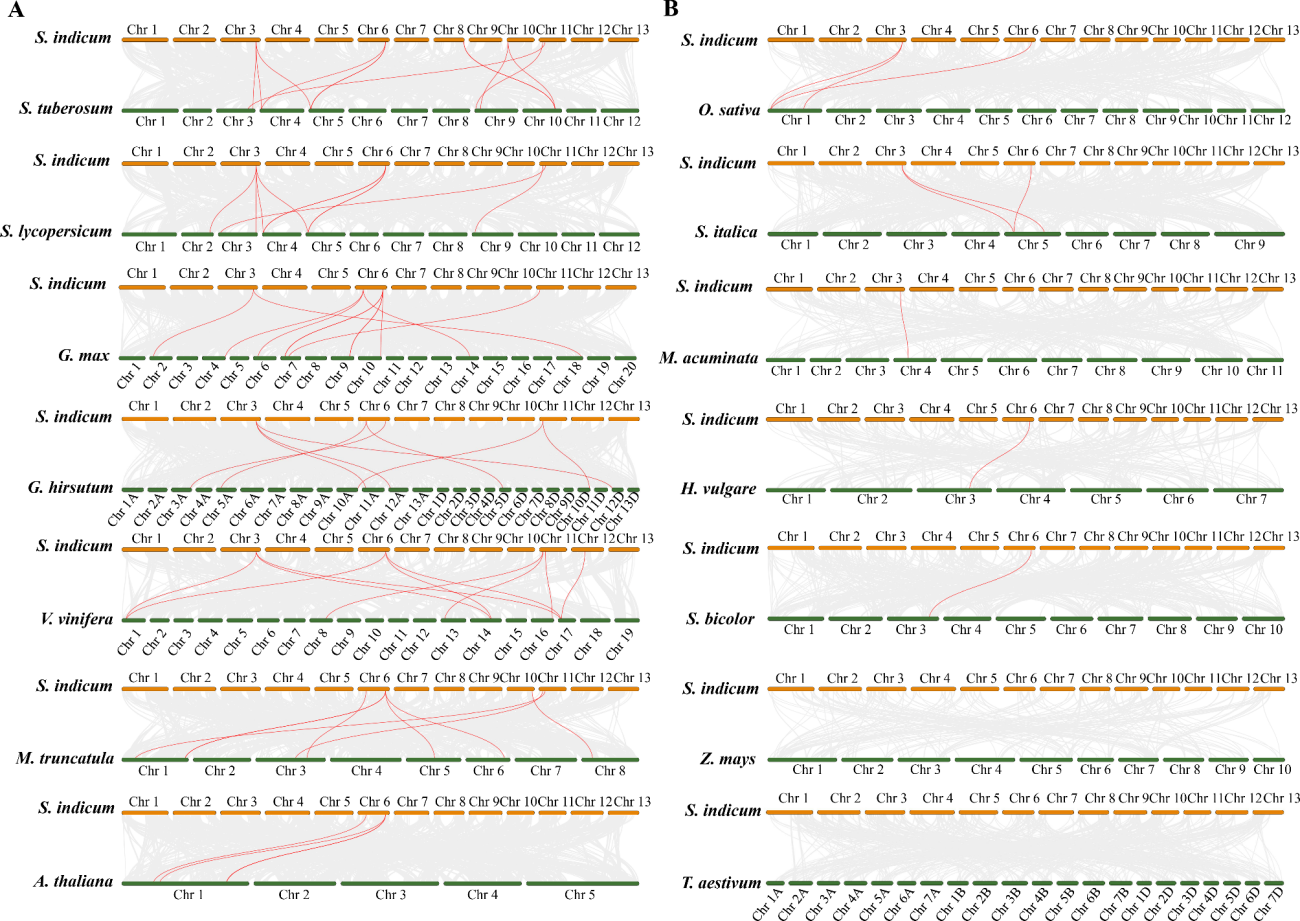
Synteny analysis of *WAKL* genes between *S. indicum* and other plant species. Orange boxes represent chromosomes of sesame while green boxes represent chromosomes of other plant species. The gray lines indicate all the syntenic gene pairs between *S. indicum* and other plant species while red lines highlight the *SiWAKL* gene pairs. (**A**) Synteny analysis of *WAKL* genes between *S. indicum* and dicotyledonous plants. (**B**) Synteny analysis of *WAKL* genes between *S. indicum* and monocotyledonous plants

To infer the syntenic relationship of *SiWAKL* genes in several plants, seven dicotyledons (*Solanum tuberosum*, *Solanum lycopersicum*, *Glycine max*, *Gossypium hirsutum*, *Vitis vinifera*, *Medicago truncatula* and *Arabidopsis thaliana*) (Fig. 5A) and seven monocotyledons (*Oryza sativa*, *Setaria italica*, *Musa acuminata*, *Hordeum vulgare*, *Sorghum bicolor*, *Zea mays* and *Triticum aestivum*) (Fig. 5B) were used for evolution analysis with *S. indicum*. The *WAKL* genes are homologous to genes in the dicotyledonous reference plants, and the number of homologous *WAKL* genes is 8 (*S. tuberosum*), 7 (*S. lycopersicum*), 5 (*G. max*), 5 (*G. hirsutum*), 6 (*V. vinifera*), 6 (*M. truncatula*) and 3 (*A. thaliana*). Nonetheless, only 2 (*O. sativa*), 2 (*S. italica*), 1 (*M. acuminata*), 1 (*H. vulgare*), 1 (*S. bicolor*), 0 (*Z. mays*) and 0 (*T. aestivum*) homologous *WAKL* genes existed in monocotyledons. More homologous *WAKL* genes were found in dicotyledons than in monocotyledons (Additional file: Table S2). In addition, *SiWAKL14* and *SiWAKL13* were homologous with 9 and 8 species, respectively, suggesting that they are crucial in the evolution of the *WAKL* gene family. (Fig. 5).

### Expression profiles of *SiWAKL* genes during *M. phaseolina* Infection

**Fig. 6:**
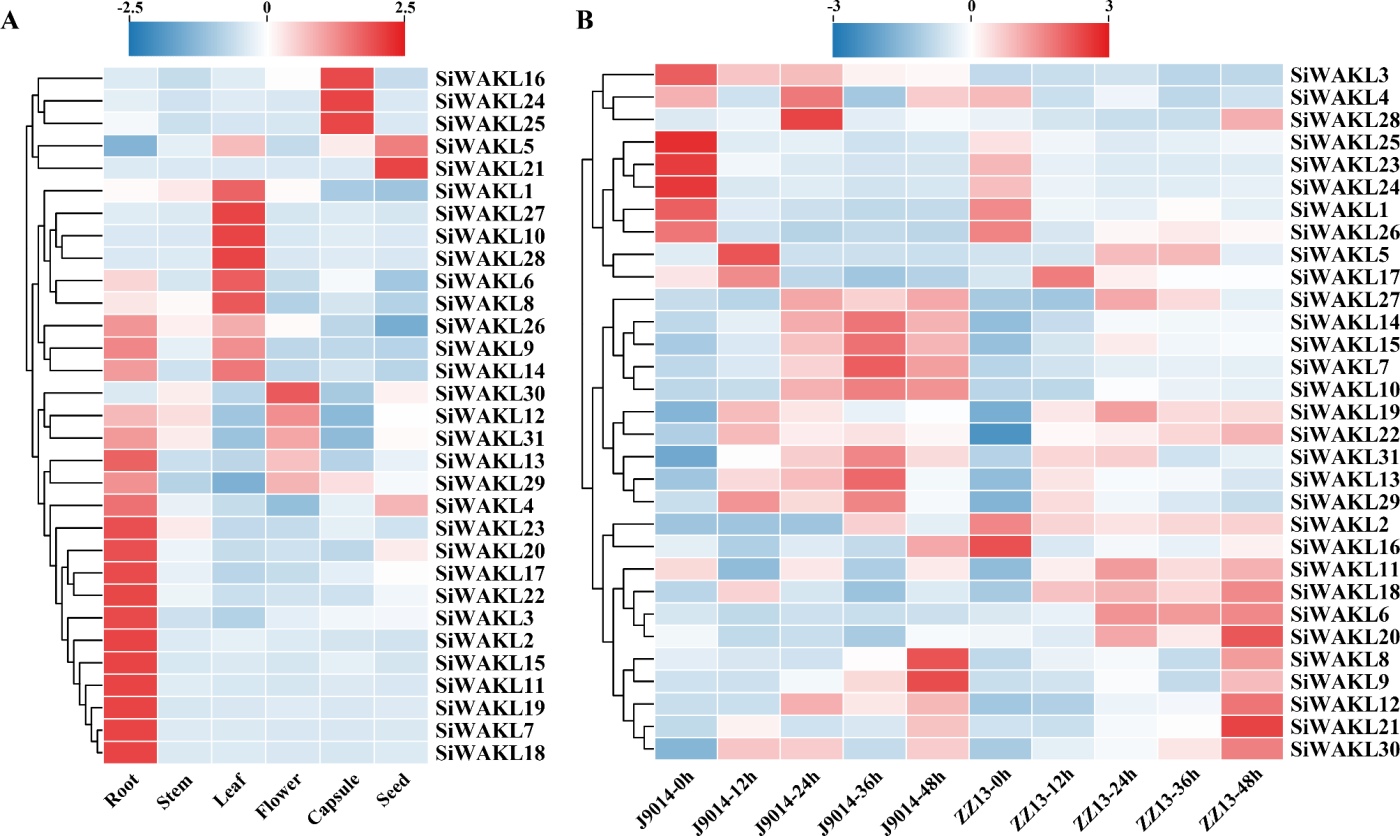
Expression profiles of *SiWAKL* genes in sesame based on RNA-seq transcriptomic analysis. FPKM values of *SiWAKL* genes were normalized. Red boxes mean higher expression level while blue boxes represent lower expression level. (**A**) Expression profiles of *SiWAKL* genes in root, stem, leaf, flower, capsule and seed tissues. (**B**) Expression profiles of *SiWAKL* genes in response to *M. phaseolina* stress within 48 h post inoculation. ZZ13, disease-resistant variety *Sesamum indicum* var. ‘Zhengzhi 13’. J9014, disease-susceptible variety *Sesamum indicum* var. ‘Ji 9014’

The expression patterns of *SiWAKL* genes can provide important clues for exploring their potential functions. To gain a broader understanding of the functions of *SiWAKLs*, the expression profiles of *SiWAKL* genes in roots, stems, leaves, flowers, capsules and seeds were analyzed using transcriptome data in this investigation. Pearson correlation analyses and principal component analyses showed that the repeatability of samples from diverse sesame tissues was good (Additional file: Figure S1A, S1B). The results revealed that different *SiWAKL* genes were expressed diversely in different tissues (Fig. 6A, Additional file: Table S3). Most *SiWAKL* genes were highly expressed in roots, followed by leaves. Among them, *SiWAKL22* and *SiWAKL24* were constitutively expressed at a high level in all tissues (Additional file: Table S3), suggesting their important role in plant growth and development. Notably, *SiWAKL6* was also highly expressed in both leaves and roots (Fig. 6A, Additional file: Table S3).

To understand the roles of the *SiWAKL* genes under *M. phaseolina* stress, the expression patterns of *SiWAKLs* were determined using transcriptome data PRJNA706471 of sesame Zhengzhi 13 (ZZ13, disease-resistant variety) and Ji 9014 (J9014, disease-susceptible variety) under *M. phaseolina* stress. The results showed that different *SiWAKL* family members responded differently to *M. phaseolina* stress and the expression of *SiWAKLs* varied with stress duration (Fig. 6B). *SiWAKL8*, *SiWAKL9*, *SiWAKL1* and *SiWAKL22* were induced by *M. phaseolina* in both ZZ13 and J9014, indicating that they may contribute to the basal resistance of sesame. In addition, *SiWAKL6*, *SiWAKL11*, *SiWAKL18*, and *SiWAKL20* were significantly induced in ZZ13 but not J9014, and their transcripts increased with stress time (Fig. 6B, Additional file: Table S4), implying that they may mediate sesame resistance to *M. phaseolina*. Although *SiWAKL20* was differentially expressed in ZZ13 versus J9014, their expression level was very low (FPKM < 1.5). *SiWAKL18* was induced in the ZZ13 within 48 h post inoculation, but it was also induced the same level in the J9014 at 12 h post inoculation. Additionally, *SiWAKL11* was induced in ZZ13 within 48 h post inoculation while it was also induced the same level in J9014 at 24 and 48 h post inoculation (Additional file: Table S4). However, the expression of *SiWAKL6* gene was induced uniquely in ZZ13 rather than J9014. The expression level of *SiWAKL6* gene increased from 1.45 to 9.99 in the resistant cultivar while that of *SiWAKL6* gene remained below 1. The overall expression level of *SiWAKL6* gene in ZZ13 is much higher than that in J9014. Therefore, we focused on *SiWAKL6* gene and cloned it, and found that the coding sequence of *SiWAKL6* was different among ZZ13 and J9014 (Additional file: Figure S2). The facts above indicated that SiWAKL6 may be related to sesame resistance to *M. phaseolina*, hence *SiWAKL6* was selected for follow-up functional characterization.

### *SiWAKL6* was induced by *M. phaseolina* and SA

**Fig. 7:**
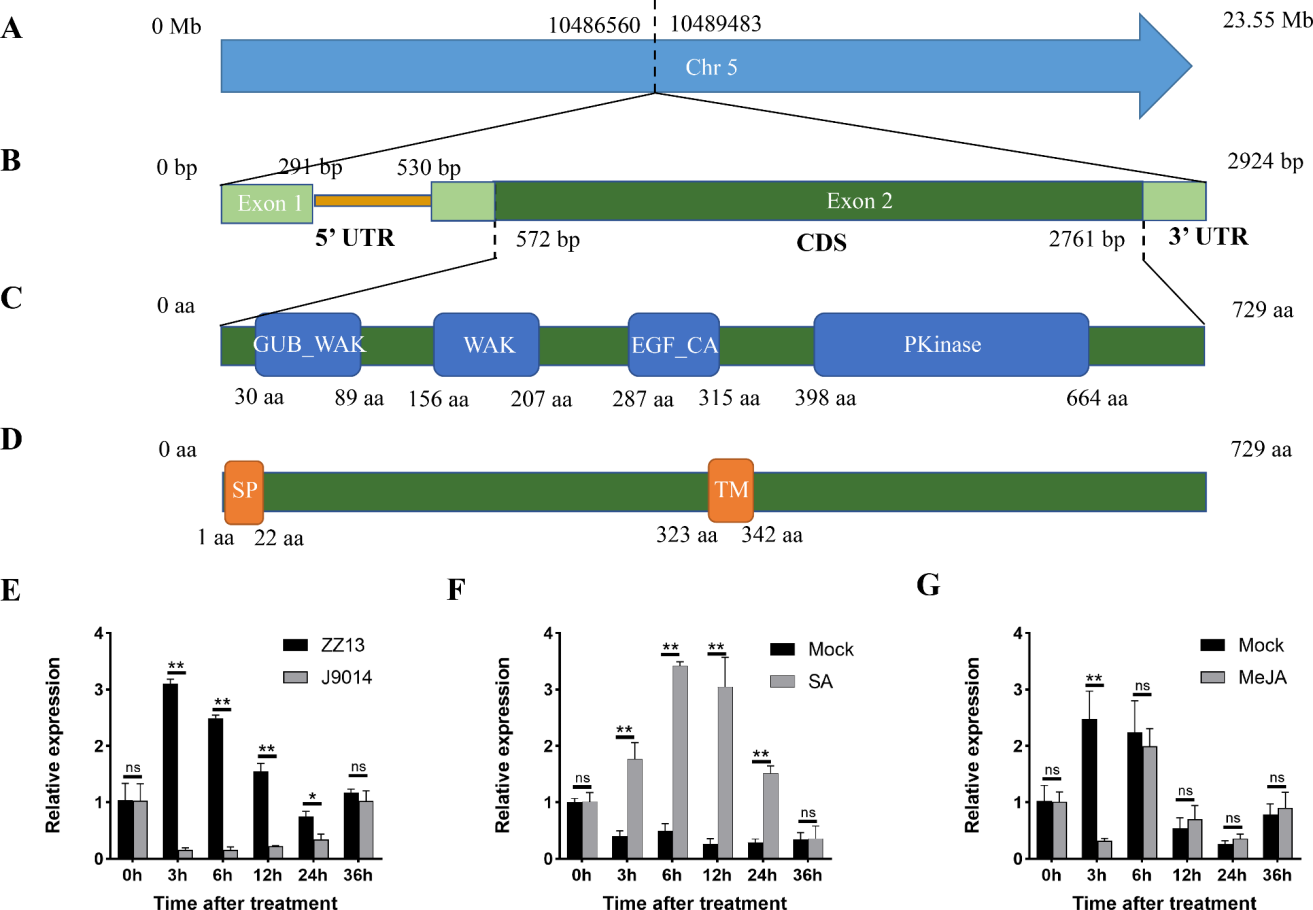
Bioinformatics and expression characteristics of SiWAKL6. The chromosomal location (**A**), gene structure (**B**), domains (**C**), signal peptide and transmembrane structure (**D**) of SiWAKL6. (**E**) The relative expression level of the *SiWAKL6* gene in ZZ13 and J9014 roots post inoculation with *M. phaseolina.* (**F**) The relative expression level of the *SiWAKL6* gene after SA treatment, with water treatment as a mock. (**G**) The relative expression level of the *SiWAKL6* gene after MeJA treatment, with water treatment as a mock. *SiWAKL6* expression were quantified with *SiUBQ5* as a normalization control. Student’s t test (*, P < 0.05; **, P < 0.01) was used to analyze the data, with three biological replicates per sample; data are the mean ± SD

To further investigate the function of *SiWAKL6* in sesame resistance to *M. phaseolina*, the complete coding sequence of *SiWAKL6* was obtained using root RNA of ZZ13. *SiWAKL6* is a 2924-bp gene located on Chr 5 (Fig. 7A) that contains two exons and one intron (Fig. 7B). The *SiWAKL6* gene encodes a 729-residue protein with a protein kinase domain in C-terminal (aa 398 to aa 664) and a GUB-WAK-bind domain in the N-terminal (aa 30 to aa 89), which is a characteristic domain of the WAKL family (Fig. 7C). A 22-residue signal peptide was detected at the N-terminal of SiWAKL6 and a transmembrane structure was predicted from aa 323 to aa 342 of SiWAKL6 (Fig. 7D). All these features are consistent with the WAKL identity of SiWAKL6.

The relative expression level of the *SiWAKL6* gene in ZZ13 and J9014 roots post inoculation with *M. phaseolina* was performed by qPCR. The results showed that *SiWAKL6* could be significantly induced by *M. phaseolina* in ZZ13, reaching a peak after 3 h. However, *SiWAKL6* was not induced in J9014, suggesting that *SiWAKL6* might regulate the high resistance of ZZ13 to *M. phaseolina* (Fig. 7E). Phytohormones commonly regulate the expression of plant disease-associated proteins [25]. To investigate the potential role of *SiWAKL6* in the plant hormonal signaling pathway, the expression pattern of *SiWAKL6* in ZZ13 after SA and MeJA treatments was examined, with water treatment as a Mock. The results showed that phytohormones affected *SiWAKL6* expression. *SiWAKL6* rapidly increased under exogenous SA treatment (Fig. 7F). However, the expression of *SiWAKL6* after exogenous MeJA treatment was similar to that after water treatment (Fig. 7G). Taken together, the results demonstrated that *SiWAKL6* was induced by *M. phaseolina* and SA, implying that *SiWAKL6* might enhance sesame resistance to *M. phaseolina* through the SA pathway.

### *SiWAKL6* enhanced *A. thaliana* resistance to *M. phaseolina* through the SA pathway

**Fig. 8:**
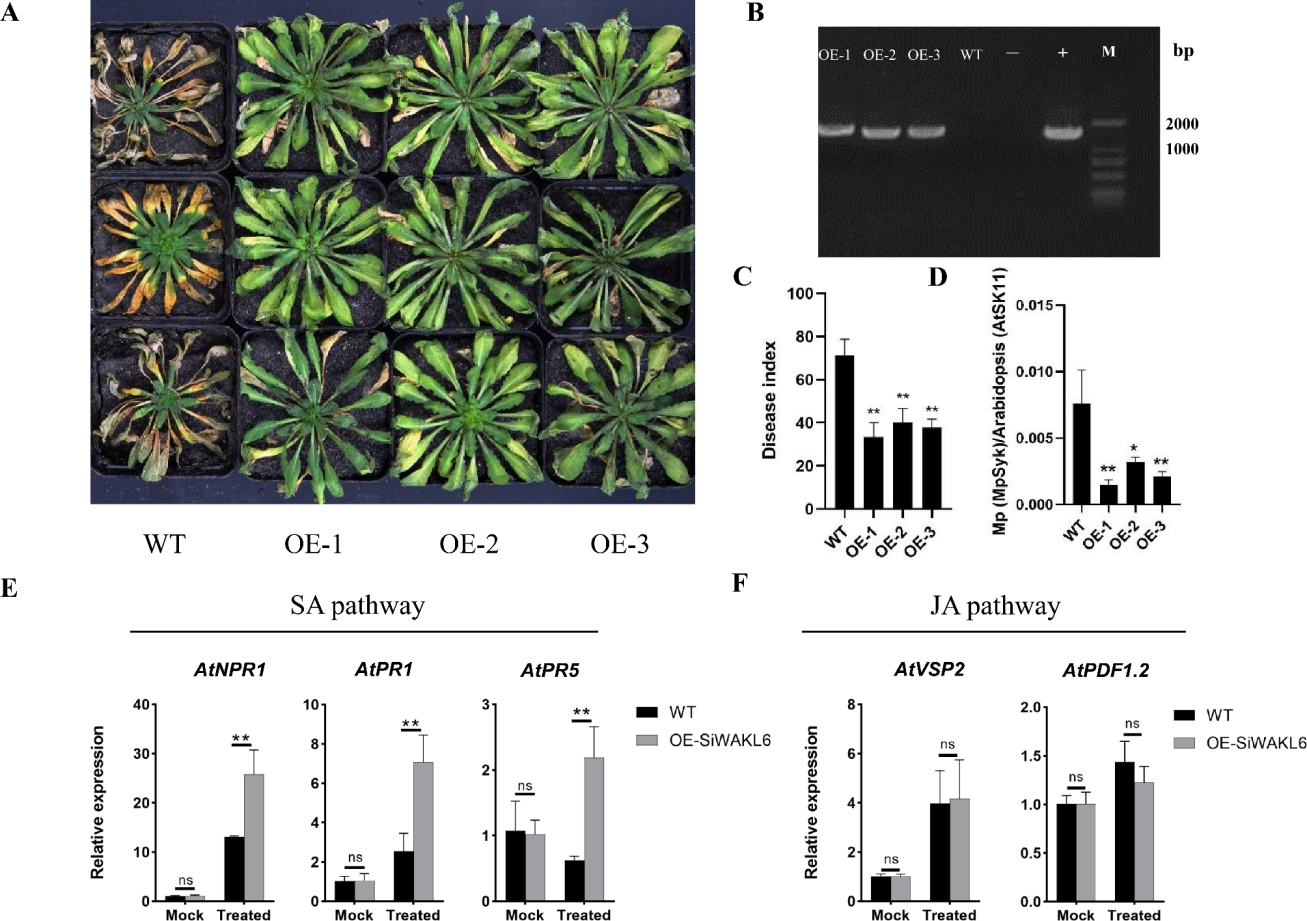
*SiWAKL6* enhanced transgenic *A. thaliana* resistance to *M. phaseolina.* (**A**) Phenotypes of WT and transgenic *A. thaliana* plants at 14 days after inoculation by *M. phaseolina*. (**B**) Transgenic plants were confirmed by PCR. “-” represents water, while “+” represents recombinant plasmid. (**C**) Disease index of WT and transgenic *A. thaliana* plants at 14 days after inoculation by *M. phaseolina*. (**D**) Relative biomass of *M. phaseolina* (MpSyk) and *A. thaliana* (AtSK11) DNA in WT and transgenic *A. thaliana* plants at 14 days after inoculation by *M. phaseolina*. (**E**) Relative expression of AtNPR1, AtPR1 and AtPR5 genes in the SA pathway at 12 h postinoculation by *M. phaseolina*, with water treatment as a mock. (**F**) Relative expression of AtVSP2 and AtPDF1.2 genes in the JA pathway at 12 h postinoculation by *M. phaseolina*, with water treatment as a mock

To further determine the function of *SiWAKL6* in resistance to *M. phaseolina*, transgenic *A. thaliana* plants overexpression *SiWAKL6* (OE-SiWAKL6) were constructed. DNA from three OE-SiWAKL6 transgenic *A. thaliana* lines (OE-1, OE-2 and OE-3) and WT (Fig. 8A) were used for PCR (Fig. 8B). Eight-week-old *A. thaliana* were inoculated with *M. phaseolina*. The results showed that the WT exhibited significant leaf chlorosis, necrosis and growth retardation, while the OE-1, OE-2 and OE-3 lines exhibited milder symptoms (Fig. 8A). Additionally, the disease index (DI) of *A. thaliana* plants was determined and found that the DI of OE-1, OE-2 and OE-3 decreased by 53%, 44% and 47%, respectively, compared with that of WT (Fig. 8C). Moreover, qPCR was performed with primer pairs specifically targeting species-specific sequence characterized amplified regions of *M. phaseolina* (*MpSyk*) and *A. thaliana* (*AtSK11*) DNA to compare the relative biomass of *M. phaseolina* and *A. thaliana*. The results showed that the relative abundance of *M. phaseolina* was reduced by 81%, 58% and 72% in the OE1, OE2 and OE3 lines, respectively, compared with the WT (Fig. 8D). The results above all implied that *SiWAKL6* can enhance transgenic *A. thaliana* resistance to *M. phaseolina*.

When *M. phaseolina* is challenged, we hypothesized that *SiWAKL6* might enhance *A. thaliana* resistance by regulating the expression of biotic stress marker genes downstream. Thus, the expression of marker genes in the SA and JA hormone signaling pathways was detected, including the *AtNPR1*, *AtPR1* and *AtPR5* genes in the SA pathway and the *AtVSP2* and *AtPDF1.2* genes in the JA pathway. The results showed that the expression of the *AtNPR1*, *AtPR1* and *AtPR5* genes was higher in OE-SiWAKL6 plants than in WT plants under *M. phaseolina* stress (Fig. 8E) while the *AtVSP2* and *AtPDF1.2* genes showed similar expression patterns in OE-SiWAKL6 and WT plants (Fig. 8F), suggesting that *SiWAKL6* could increase *A. thaliana* resistance by regulating genes in the SA pathway.

### *SiWAKL6* reconstructed ROS homeostasis in plant immunity

Under biotic stress, plant immunity depends on the production of ROS in plants, but the excess accumulation of ROS causes oxidative damage. Meanwhile, the antioxidant system in plants initiates the synthesis of superoxide dismutase (SOD), catalase (CAT) and peroxidase (POD) to scavenge ROS in plants and inhibit cell death. To investigate whether *SiWAKL6* gene-mediated resistance to *M. phaseolina* was associated with ROS homeostasis, we analyzed the relative ROS levels (hydrogen peroxide (H_2_O_2_) and malonaldehyde (MDA) and relative antioxidant enzyme activities (SOD and CAT) in WT and transgenic plants (OE-SiWAKL6) at 14 days after inoculation with *M. phaseolina*.

**Fig. 9:**
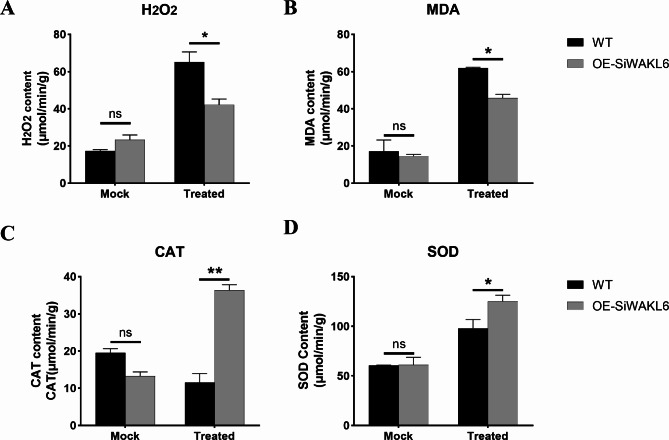
*SiWAKL6* regulated ROS homeostasis in resistance to *M. phaseolina.* H_2_O_2_ (**A**) and MDA (**B**) contents in WT and OE-SiWAKL6 plants 14 days after inoculation by *M. phaseolina*, with water treatment as a mock. CAT (**C**) and SOD (**D**) enzyme activity in WT and OE-SiWAKL6 plants 14 days after inoculation by *M. phaseolina*, with water treatment as a mock

We found that the concentrations of H_2_O_2_ and MDA were increased in both OE-SiWAKL6 and WT plants under *M. phaseolina* stress. However, WT accumulated more H_2_O_2_ (Fig. 9A) and MDA (Fig. 9B) than OE-SiWAKL6. The high H_2_O_2_ and MDA levels in WT could cause hypersensitivity in the *M. phaseolina* infection process, which provides insight into a potential link between ROS levels and *M. phaseolina* resistance. In addition, the CAT (Fig. 9C) and SOD (Fig. 9D) activities were enhanced in OE-SiWAKL6 compared with WT plants post inoculation by *M. phaseolina*, suggesting *SiWAKL6* was involved in plant immunity by reconstructing ROS homeostasis, which was regulated by an active antioxidant system.

## Discussion

WAKL proteins, acting as a link between the cell wall and plasma membrane, enable plants to sense external signals, which is vital for plant growth, development and response to stress. The *WAKL* gene family has been reported successively in *A. thaliana* (27 WAKLs) [9], *Oryza sativa* (130 WAKLs) [26], *Gossypium hirsutum* (99 WAKLs) [27], *Brassica rapa* ssp. *pekinensis* (96 WAKLs) [28] and *Populus trichocarpa* (175 WAKLs) [29] genomes. However, there is no systematic analysis of the *WAKL* gene family in sesame, and the functions of the *SiWAKL* genes are still unclear. The publication of high-quality whole genome sequences of sesame provided the possibility for sesame transcriptome sequencing and gene family identification [30, 31]. In this study, a total of 31 *SiWAKL* genes were identified within the sesame genome, which are distributed on 8 chromosomes. SiWAKL proteins all comprise GUB-WAK-bind domains and protein kinase domains. Compared to monocotyledons (130 WAKLs in rice, 91 WAKLs in barley and 99 WAKLs in cotton), the WAKL gene family is generally smaller in dicotyledons (31 WAKLs in sesame, 27 WAKLs in *Arabidopsis* and 21 WAKLs in sweet orange), indicating that *WAKL* genes have undergone different degrees of expansion during evolution between monocotyledons and dicotyledons.

Within the sesame genome, 18 *SiWAKL* genes form ten tandem duplication events while 2 *SiWAKL* genes constitute one segmental duplication event. The tandem duplication events were much more than the segmental duplication events, indicating that tandem duplication events were the principal factor in the expansion of the *SiWAKL* gene family in this study (Fig. 4). In addition, collinearity analysis showed that the homologous *WAKL* genes existed much more in dicotyledons than monocotyledons (Fig. 5, Additional file: Table S2), implying that the duplication of the *WAKL* gene probably occurred after the differentiation of dicotyledons and monocotyledons. Sesame species were evolutionarily more closely related to potato species and tomato species [31]. Interestingly, *SiWAKL* genes had the most homologous gene pairs with those in potato and tomato, suggesting that *WAKL* genes in these species may have a common ancestor. The evidence above provided clues to investigate the evolutionary process of WAKL genes in sesame.

The WAKL family is a crucial class of pattern recognition receptors that function in recognizing pathogens in plants. As shown by GO annotation (Fig. 3), SiWAKLs located at the plasma membrane could bind to polysaccharides, ATP and Ca^2+^. This is consistent with previous studies in other species, such as the *OsWAK1* gene in rice [32], the *ZmWAK-Hnt1* gene in maize [33] and the *TaWAK-6D* gene in wheat [34], which are all localized in the plasma membrane. On the one hand, WAKLs can initiate plant immune responses by binding oligogalacturonides (OGs) and pectins. They can perceive exogenous biotic and abiotic stimuli by GUB-WAK-bind domains. On the other hand, WAKLs can transmit signals into the cell to activate downstream cascade responses by their Pkinase domains [35]. AtWAK1 and AtWAK2 have been shown to interact with OGs and pectins in vitro [36, 37]. When treated with OGs, the expression of *AtWAK1* could be induced, followed by the initiation of downstream immune responses such as callose accumulation, which enhanced *A. thaliana* resistance to *Botrytis cinerea* [38]. In this study, *SiWAKL6* responds to *M. phaseolina* and exogenous SA (Fig. 7E and F), which is similar to previous studies. *AtWAK1* also is induced by pathogen infection or exogenous SA treatment [39] to enhance plant resistance. *WAKLs* generally confer plant resistance against pathogens by regulating biological processes including cell wall reinforcement [20], activation of *PR* genes [40], SA or JA accumulation [5] and ROS homeostasis [5]. Rice *OsWAK14*, *OsWAK91* and *OsWAK92* genes can positively regulate resistance to rice blast fungus by increasing the expression of *PR* genes. In addition, *OsWAK91* is a key gene for H_2_O_2_ production in rice, suggesting that *OsWAK91* can enhance its resistance to pathogens by re-establishing ROS homeostasis and upregulating *PR* genes [41]. Additionally, it has been reported that *CsWAKL08* in citrus confers resistance to citrus bacterial canker via ROS control and JA signaling [5]. Similarly, the *SiWAKL6* gene in this study might also enhance sesame resistance to *M. phaseolina* through the SA signaling pathway and re-establishment of ROS homeostasis.

WAKL proteins can exert phosphorylation to participate in signal transduction in plants. Studies have reported that *AtWAK2* can activate mitogen-activated protein kinases 3 (*MPK3*) and *MPK6* and transmit signals intracellularly in plant innate immunity [36, 42]. Wang et al. showed that cotton *GhWAK7A* confers high resistance to *Verticillium dahliae* and *Fusarium oxysporum* f. sp *vasinfectum*. GhWAK7A can phosphorylate the chitin receptor complex upon pathogen infestation, which can activate cytoplasmic signaling pathways, including ROS production, activation of MAPK cascades and expression of *PR* genes. Moreover, silencing of the *GhWAK7A* gene impaired the activation of *GhMPK3* and *GhMPK6* genes in cotton and attenuated resistance [43]. However, it is unknown whether *SiWAKL6* can mediate downstream signaling pathways through phosphorylation and MAPK cascades, and subsequent studies will continue.

## Conclusion

In this study, a total of 31 *SiWAKL* genes were identified and analyzed for their chromosomal distribution, taxonomy, protein structures, duplication events and expression patterns. The expansion of the *SiWAKL* gene family was mainly due to tandem duplication events. Transcriptomic and qPCR analyses showed that *SiWAKL6* was a potential gene involved in sesame resistance to *M. phaseolina*, which was induced by *M. phaseolina* and exogenous SA. Further functional analysis revealed that *SiWAKL6* overexpression in transgenic *A. thaliana* plants enhanced *A. thaliana* resistance to *M. phaseolina*. We found that *SiWAKL6* conferred higher resistance to transgenic *A. thaliana* plants by increasing the expression of SA pathway related genes and reconstructing ROS homeostasis. Taken together, the results of this study provide new insight into the mechanisms of *SiWAKL6* gene acting in sesame immunity and a basis for the application of *SiWAKLs* in molecular breeding for sesame resistance to *M. phaseolina*.

## Methods

### Identification and bioinformatics analysis of the *WAKL* gene family in sesame

Gene and protein sequences of all 27 WAKLs of *A. thaliana* were downloaded from the TAIR website (https://www.arabidopsis.org/). The genome and proteome sequences of sesame were provided by the Sesame Research Center, Henan Academy of Agricultural Sciences [30, 44]. To exhaustively identify WAKLs in sesame, all 27 AtWAKL proteins were used to perform BLASTP with the sesame proteome and all candidate genes with E values less than 10^− 10^ were screened. The candidate sequences were detected in the InterPro database (https://www.ebi.ac.uk/interpro/) for the presence of both the GUB-WAK-bind domain and PKinase domain. Proteins that met all conditions were considered sesame WAKL proteins.

Multiple sequence alignment of SiWAKL proteins was analyzed using the ClustalW method. Phylogenetic analysis based on the aligned sequences of WAKL proteins was performed by MEGA 7 software [45] with the neighbor Joining (NJ) method (Bootstrap = l000). Additionally, the chromosomal location of the *SiWAKL* genes was visualized by TBtools [46]. The MCScanX [47] program was used to determine collinear orthologous gene duplications (Tandem and segmental duplications) among the sesame *WAKL* gene family and syntenic *WAKL* genes between sesame and other plant species. The genome files and annotation files of *S. tuberosum*, *G. max*, *S. lycopersicum*, *M. truncatula*, *A. thaliana*, *V. vinifera*, *G. hirsutum*, *H. vulgare*, *Z. mays*, *T. aestivum*, *O. sativa*, *M. acuminata*, *S. italica* and *S. bicolor* were downloaded from the Phytozome database [48].

The isoelectric point (*pI*) and molecular weight (MW) of SiWAKL proteins were predicted on the ExPASy website (https://web.expasy.org/compute_pi/). All SiWAKL protein sequences were submitted to the MEME online server (http://meme-suite.org/) to search for conserved motifs with the following parameters: maximum number of motifs limited to 15 and motif size limited between 6 and 50 amino acids. Gene Ontology (GO) functional annotation of SiWAKL proteins were predicted by PANNZER 2 online server (http://ekhidna2.biocenter.helsinki.fi/sanspanz/). Signal peptide and subcellular localization prediction of WAKL proteins were performed at the websites SignalP-5.0 (https://services.healthtech.dtu.dk/services/SignalP-5.0/) and WoLF PSORT (http://psort.hgc.jp/), respectively.

### Plant materials and treatment

Seeds of the disease-susceptible genotype J9014 and disease-resistant genotype ZZ13 [49] were disinfected in 5% sodium hypochlorite solution for 15 min and then in 70% alcohol for 30 s. After that, the seeds were rinsed 3–4 times with sterile water, and then dried and planted in mixed nutrient soil (sterile soil: nutrient soil: sterile vermiculite = 3:1:1). The sesame seedlings were cultured under 29 ± 1 °C, 80% relative humidity and a photoperiod of light for 16 h and dark for 8 h. Arabidopsis seeds were sterilized with 5% sodium hypochlorite solution for 10 min and repeatedly rinsed with sterile water 3–5 times. Then the seeds were sown on 1/2 MS medium and placed in a refrigerator at 4 °C for 4 days. After treatment, they were cultured in an incubator at 22 ± 1 °C with 16 h light and 8 h dark conditions. When Arabidopsis grew to two true leaves, the seedlings were transferred to mixed nutrient soil (nutrient soil: vermiculite = 3:1) and continued to be cultured under the same conditions.

The genotype ZZ13 was selected for the tissue-specific RNA-seq of sesame. Sampling methods were referenced to Dossou et al. [50]. ZZ13 was grown under normal culture conditions (16 h light/30°C and 8 h dark/28°C). Flower tissues with consistent growth were randomly sampled and the locations were marked. The capsules at the markers were sampled along with all other tissues (roots, middle stems, middle leaves, fresh capsules and fresh seeds) two weeks later for RNA extraction. When sesame capsules were removed, fresh seeds were separated from the fresh capsules on ice immediately to obtain fresh seeds and capsules. The samples used for RNA-sequencing later.

The method of inoculation with *M. phaseolina* was performed as described previously [49]. When sesame seedings grew to three pairs of true leaves, pots were irrigated with 167 mL of 200 µmol/L methyl jasmonate (MeJA) and 2 µmol/L salicylic acid (SA) solutions were irrigated to each pot for treatment, respectively, with 167 mL water treatment as a control (Mock). Sesame root tissues were collected at 0 h, 3 h, 6 h, 12 h, 24 and 36 h after treatment and stored at -80 °C.

### Total RNA extraction and cDNA library construction

Purity and concentration of total RNA of sesame different tissues of ZZ13 extracted with the TransZol Up Plus RNA Kit were examined by spectrophotometer NanoDrop 2000 while the integrality of total RNA detected by Agient2100/LabChip GX. Then, the cDNA library was constructed. After the library constructed, the initial quantification was performed by the Qubit 3.0 fluorescence quantification instrument. Subsequently, the insert fragment of cDNA library was detected by Qsep400 high-throughput system while the effective concentration of the cDNA library (> 2 nM) was measured by Q-PCR. After quality control of the cDNA library, PE150 sequencing was performed using Illumina NovaSeq6000.

### Gene expression analysis

To understand the expression patterns of *WAKL* genes involved in sesame resistance to *M. phaseolina* stress, the tissue-specific transcriptome data PRJNA892254 of ZZ13 and the transcriptome data PRJNA706471 [49] of sesame and *M. phaseolina* interactions were used in this study. “PRJNA892254” is the transcriptome data of root, stem, leaf, flower, capsule and seed tissue of ZZ13 under normal conditions. “PRJNA706471” is the transcriptome data of ZZ13 and J9014 at 0 h, 12 h, 24 h, 36 and 48 h postinoculation with *M. phaseolina*.

To standardize the gene expression levels of each sample, the clean reads were converted into fragments per kilobase of exon model per million mapped reads (FPKM) [51]. The number of reads of each gene was counted by StringTie (version: 1.3.0) and then clean reads were mapped to the sesame genome with HISAT2 (version: 2.0.4) [52, 53]. Finally, the FPKM value of each gene was calculated by the trimmed mean of M values method [54].

The sesame reference genome is provided by the Sesame Research Center, Henan Academy of Agricultural Sciences [30, 44].

### Construction of the overexpression plasmids

The full length *SiWAKL6* coding sequence was cloned from the varieties ZZ13 and J9014. *SiWAKL6* in ZZ13 was inserted into pCambia2301 plasmids using homologous recombination to construct a 35 S::SiWAKL6 overexpression vector. The 35 S::SiWAKL6 recombinant vector was transformed into wild-type (WT) *A. thaliana* plants (Col-0) mediated by *Agrobacterium tumefaciens* GV3101. The transgenic plants were screened on 1/2 MS medium containing 50 mg/L kanamycin, and the forward primer was designed based on the vector sequence upstream of the promoter and reverse primers were designed downstream of the *SiWAKL6* gene for PCR identification of the transgenic plants. Three independent T3 transgenic lines were used for subsequent experiments. The primers were 35 S-F (GACGCACAATCCCACTATCC) and SiWAKL6-R (TTGGTTCATGGATGTGTCGG).

### Analysis of *M. phaseolina* resistance in transgenic *A. thaliana* plants

Strain *M. phaseolina* was inoculated in PDA solid medium and incubated at 30 °C for 7 days until the mycelium covered the petri dishes. Then, the mycelium was divided into blocks, and each 1/2 petri dish of strains was inoculated evenly into 300 mL of PD liquid medium and incubated at 30 °C and 200 r/min for 5 days. Subsequently, the mycelial suspension was obtained by breaking up the solution with a tissue masher. Each 20 mL mycelium suspension was mixed with 100 mL sterilized water and 120 g sterilized stroma (nutritional soil: vermiculite = 3:1). The WT and transgenic *A. thaliana* were transplanted to the fungal soil when they had grown for eight weeks. The leaf tissue was taken 12 h after inoculation and stored at -80 °C.

Plant disease classes were classified using a scale of 0–5 based on the phenotypes of leaf chlorosis and necrosis with reference to criteria from a previous study [55]. And the formula of the disease index (DI) is as follows.

Disease index (DI) = Σ (Number of diseased plant each level × The value of each level) / (Total number of the investigated plants × The value of the highest level) × 100.

### Analysis of qPCR and ROS contents

In sesame, RNA extraction, cDNA synthesis and qPCR were performed on sesame root tissues after different treatments. In *A. thaliana*, RNA extraction, cDNA synthesis and qPCR were performed on leaf tissues post inoculation with *M. phaseolina.* Relative expression levels of genes in sesame and *A. thaliana* were quantified by the CFX 384™ real-time system made in Singapore and the 2× ChamQ Universal SYBR qPCR Master Mix (Vazyme, Nanjing, China) with the 2^−ΔΔCt^ method. Each sample had 3 replicates. The relative expression levels of sesame genes were normalized to that of the *SiUBQ5* gene. The relative expression levels of *A. thaliana* genes were normalized to that of the *AtUBQ10* gene. The primers for qPCR are shown in Additional file: Table S5.

Fourteen days post inoculation with *M. phaseolina*, the leaf tissues of *A. thaliana* plants were taken. The contents of H_2_O_2_ and MDA as well as the activities of CAT and SOD were detected using kits (Grace Biotechnology, Suzhou, China).

### Electronic supplementary material

Below is the link to the electronic supplementary material.

Supplementary Material 1

Supplementary Material 2

Supplementary Material 3

Supplementary Material 4

Supplementary Material 5

Supplementary Material 6

Supplementary Material 7

Supplementary Material 8

## Data Availability

Data is available at NCBI SRA accession: PRJNA892254 and PRJNA706471.
