# Supplementary figures and images for: Genome-wide characterization of the wall-associated kinase-like (WAKL) family in sesame (Sesamum indicum) identifies a SiWAKL6 gene involved in resistance to Macrophomina Phaseolina
Source: BMC Plant Biol. 2023 Dec 7;23:624. doi: 10.1186/s12870-023-04658-1 (PMC10702004; doi:10.1186/s12870-023-04658-1)

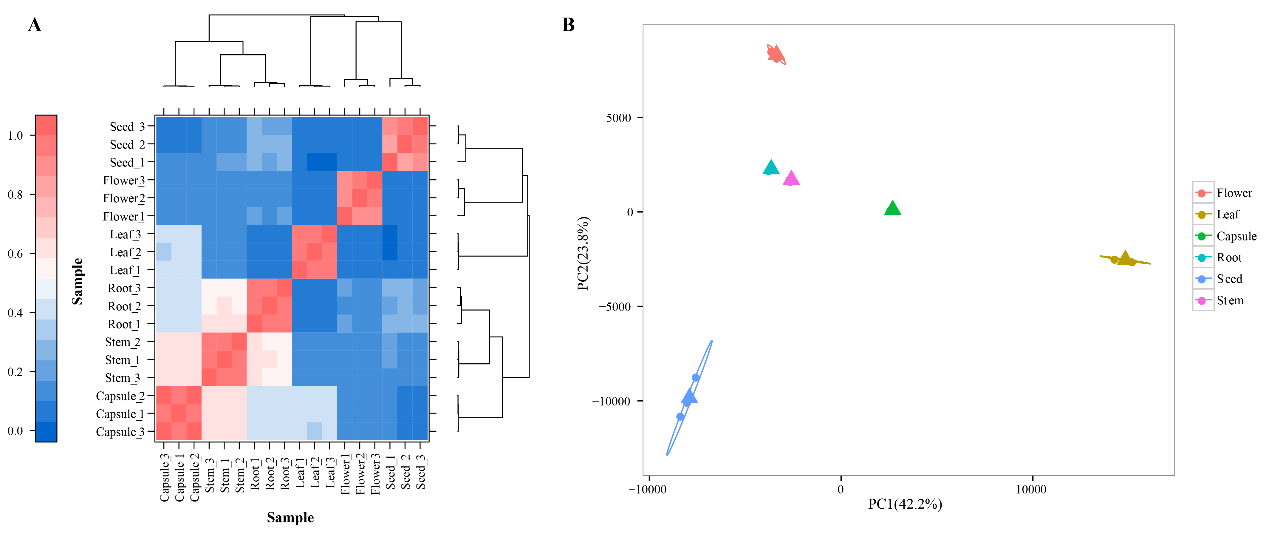


**Figure S1.** Pearson correlation coefficients (A) and principal component analyses (B) of 18 samples.

Supplement: Supplementary file 1 — Supplementary Material 1 [file 12870_2023_4658_MOESM1_ESM.docx]

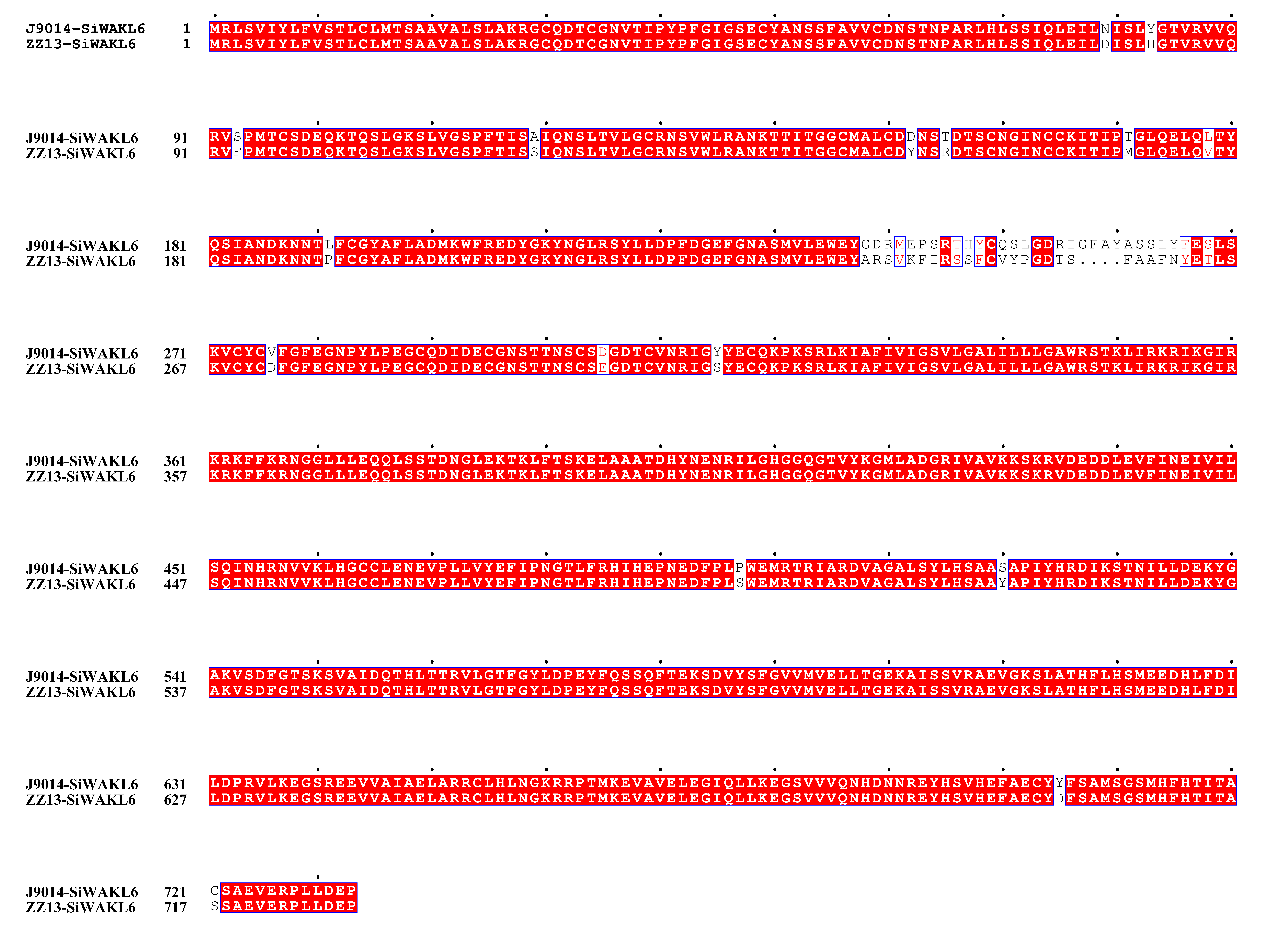


**Figure S2.** Alignment of sequences of SiWAKL6 in J9014 and ZZ13.

Supplement: Supplementary file 2 — Supplementary Material 2 [file 12870_2023_4658_MOESM2_ESM.docx]
