# Supplementary material for: Genome-wide characterization of the wall-associated kinase-like (WAKL) family in sesame (Sesamum indicum) identifies a SiWAKL6 gene involved in resistance to Macrophomina Phaseolina
Source: BMC Plant Biol. 2023 Dec 7;23:624. doi: 10.1186/s12870-023-04658-1 (PMC10702004; doi:10.1186/s12870-023-04658-1)
Supplement: Supplementary file 8 — Supplementary Material 8 [file 12870_2023_4658_MOESM8_ESM.docx]

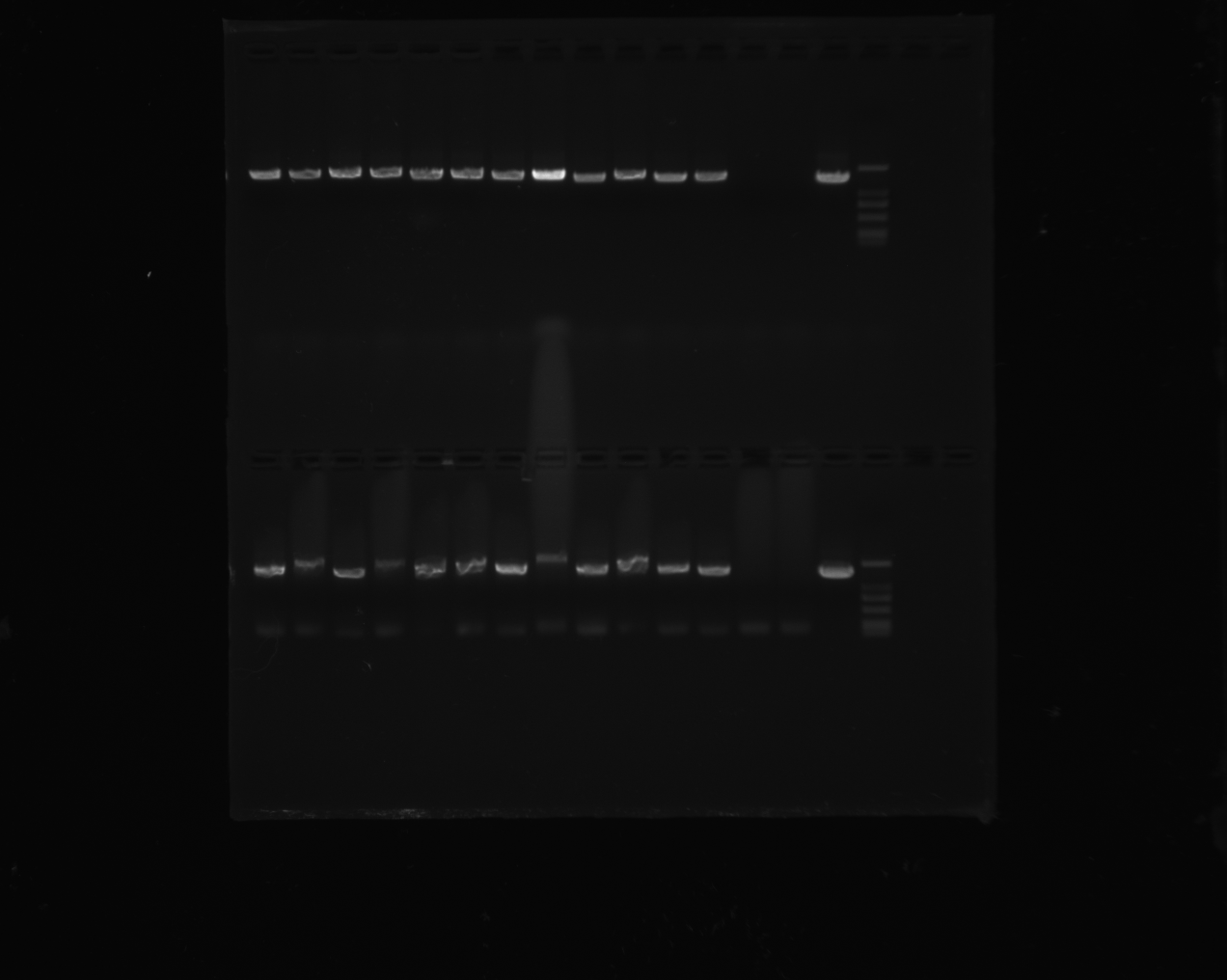

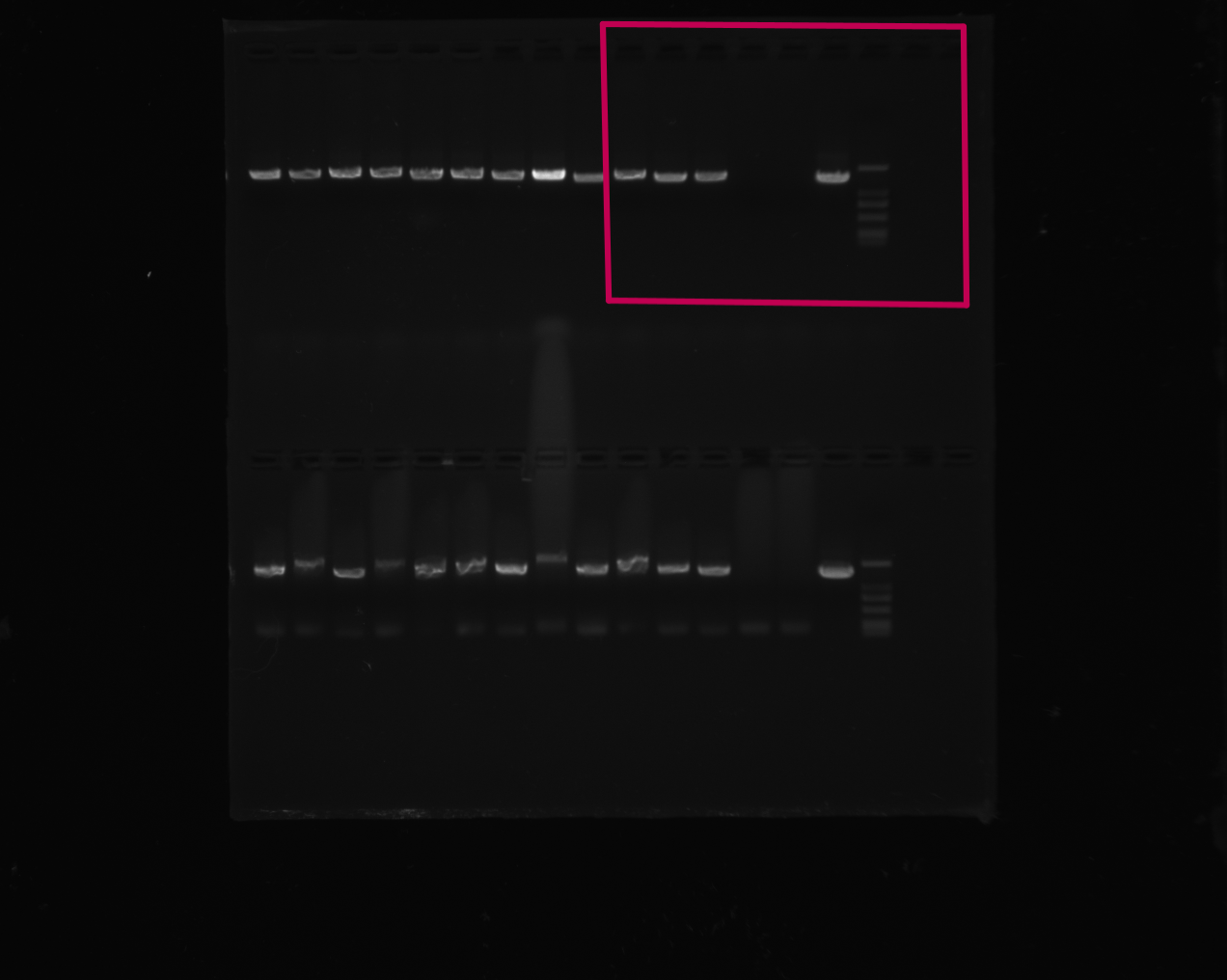


**Figure 8B**. Full length membranes of OE-SiWAKL6 transgenic plants were confirmed by PCR (Left) and the cropped area has been highlighted (Right).
